# Supplementary material for: Effect of a Screening and Education Programme on Knowledge, Beliefs, and Practices Regarding Osteoporosis among Malaysians
Source: Int J Environ Res Public Health. 2022 May 17;19(10):6072. doi: 10.3390/ijerph19106072 (PMC9141639; doi:10.3390/ijerph19106072)
Supplement: Supplementary file 1 [file ijerph-19-06072-s001.zip › ijerph-1697326-supplementary.pdf]

Table S1. The distribution of responses to osteoporosis knowledge questions of subjects.

Items	Answer	Responses		
		**True, N(%)**	**False, N (%)**	**Don't know, N (%)**
**General knowledge regarding osteoporosis**				
Makes bone become brittle and weak from loss of tissue, thus more likely to break (fracture).	True	378 (94.5)	11 (2.8)	11 (2.8)
Osteoporosis will result in knee pain.	False	265 (66.3)	75 (18.8)	60 (15.0)
Postmenopausal women have no risk to develop osteoporosis.	False	11 (2.8)	308 (77.0)	81 (20.3)
Osteoporosis is an untreatable disease.	False	77 (19.3)	263 (65.8)	60 (15.0)
Osteoporosis can be diagnosed by measuring bone mineral density.	True	349 (87.3)	8 (2.0)	43 (10.8)
I do not need to do bone mineral density test unless I fracture my bones.	False	55 (13.8)	334 (83.5)	11 (2.8)
**Prevention knowledge regarding osteoporosis**				
Consume calcium supplements help me to prevent osteoporosis.	True	348 (87.0)	33 (8.3)	19 (4.8)
The regular intake of calcium supplements can lead to formation of kidney stones.	False	197 (49.3)	55 (13.8)	148 (37.0)
Foods such as milk, tofu, yellow dhal and spinach are rich in calcium.	True	373 (93.3)	6 (1.5)	21 (5.3)
By exposing my skin to sunlight for about 15 minutes a day, I can obtain my recommended daily intake of vitamin D.	True	240 (60.0)	100 (25.0)	60 (15.0)
Intake of glucocorticoids may increase the risk of osteoporosis.	True	139 (34.8)	32 (8.0)	229 (57.3)
I must have good vision and comfortable shoes with good grip to prevent me from falling.	True	363 (90.8)	34 (8.5)	3 (0.8)

**T* True, *F* False, *DK* Don't Know

Table S2. Response to the Osteoporosis Health Belief Scale among the subjects

Items	Health Belief Scale				
	SD, N (%)	D, N (%)	N, N (%)	A, N (%)	SA, N (%)
Makes bone become brittle and weak from loss of tissue, thus more likely to break (fracture).	3 (0.8)	95 (23.8)	89 (22.3)	188 (47.0)	25 (6.3)
Osteoporosis will result in knee pain.	7 (1.8)	251 (62.8)	47 (11.8)	90 (22.5)	5 (1.3)
Postmenopausal women have no risk to develop osteoporosis.	1 (0.3)	79 (19.8)	46 (11.5)	225 (56.3)	49 (12.3)
Osteoporosis is an untreatable disease.	2 (0.5)	15 (3.8)	21 (5.3)	305 (76.3)	57 (14.3)
Osteoporosis can be diagnosed by measuring bone mineral density.	-	19 (4.8)	39 (9.8)	300 (75.0)	42 (10.5)
I do not need to do bone mineral density test unless I fracture my bones.	18 (4.5)	239 (59.8)	44 (11.0)	97 (24.3)	2 (0.5)
Consume calcium supplements help me to prevent osteoporosis.	11 (2.8)	238 (59.5)	52 (13.0)	96 (24.0)	3 (0.8)
The regular intake of calcium supplements can lead to formation of kidney stones.	7 (1.8)	298 (74.5)	42 (10.5)	49 (12.3)	4 (1.0)
Foods such as milk, tofu, yellow dhal and spinach are rich in calcium.	21 (5.3)	330 (82.5)	35 (8.8)	13 (3.3)	1 (0.3)
By exposing my skin to sunlight for about 15 minutes a day, I can obtain my recommended daily intake of vitamin D.	-	45 (11.3)	55 (13.8)	277 (69.3)	23 (5.8)
Intake of glucocorticoids may increase the risk of osteoporosis.	-	83 (20.8)	44 (11.0)	254 (63.5)	19 (4.8)
I must have good vision and comfortable shoes with good grip to prevent me from falling.	-	4 (1.0)	38 (9.5)	322 (80.5)	36 (9.0)

**SD* Strongly Agree, *D* Disagree, *N* Neutral, *A* Agree, *SA* Strongly Agree

Table S3. Barriers to achieving optimal bone health

Reasons	Low Calcium Supplement Intake Men (n=135)	Women (n=122)	Overall (n=257)
Calcium supplement is expensive	1	1	2
Calcium supplement causes kidney stone formation	1	-	1
Don't feel the need	67	71	138
Favour to get calcium from food sources	65	49	114
Calcium supplement causes constipation	1	1	2
**Reasons**	**Low dairy product intake Men (n=109)**	**Women (n=94)**	**Overall (n=202)**
Dairy products are expensive	3	1	4
Aversion to the taste of milk	10	32	42
Not a habit	93	53	146
Dairy products are fattening	1	5	6
Lactose intolerance	2	2	4
**Reasons**	**Rarely exercise Men (n=65)**	**Women (n=77)**	**Overall (n=142)**
Health problem (swollen leg or knee pain)	-	4	4
No companion	-	1	1
Busy with work/house chore	65	72	137

Table S4. Number of referrals during the follow-up.

Status	Referral Information		
	Men	Women	Overall
**Discovered as osteoporosis**	10	33	43
**Refused referral**	-	9	9
**Requested for referral letter but did not meet doctors**	7	9	16
**Did not come back for follow up**	1	5	6
**Met doctors with the referral letter**	3	15	18
**% successful referral**	41.86%		

Table S5. Reasons for not meeting medical doctors for further consultation.

Reasons	Not Going to Meet Dr		
	**Men (n=7)**	**Women (n=9)**	**Overall (n=16)**
**Lifestyle changes at home (example: exercise or diet)**	1	5	6
**Busy with work/class**	5	2	7
**Fear of taking medication**	1	2	3

Table S6. Treatment given to the subjects and their compliance.

Treatment Given	Compliance	Met Medical Doctors with The Referral Letter		
		Men (n=3)	Women (n=15)	Overall (n=18)
A combination of alendronate sodium 91.37 mg (eq to 70 mg anhydrous free acid) and cholecalciferol 140 µg (eq to 5600 IU vitamin D3)	Once a week	2	6	8
Calcium supplement	Once daily	-	4	5
	2-3 times a week	-	1	1
Lifestyle changes without medication		1	4	5
Followed prescription accordingly		3	15	18
